# Apathy in rapid eye movement sleep behaviour disorder is associated with serotonin depletion in the dorsal raphe nucleus

**DOI:** 10.1093/brain/awy240

**Published:** 2018-09-12

**Authors:** Thomas R Barber, Ludovica Griffanti, Kinan Muhammed, Daniel S Drew, Kevin M Bradley, Daniel R McGowan, Marie Crabbe, Christine Lo, Clare E Mackay, Masud Husain, Michele T Hu, Johannes C Klein

**Affiliations:** 1 Oxford Parkinson’s Disease Centre, Oxford, UK; 2 Nuffield Department of Clinical Neurosciences, University of Oxford, Oxford, UK; 3 Oxford Centre for Human Brain Activity, Wellcome Centre for Integrative Neuroimaging, Department of Psychiatry, University of Oxford, Oxford, UK; 4 Oxford Centre for Functional MRI of the Brain, Wellcome Centre for Integrative Neuroimaging, Nuffield Department of Clinical Neurosciences, University of Oxford, Oxford, UK; 5 Department of Experimental Psychology, University of Oxford, Oxford, UK; 6 Department of Radiology, Churchill Hospital, Oxford, UK; 7 Radiation Physics and Protection Department, Churchill Hospital, Oxford, UK

**Keywords:** apathy, parkinsonism, RBD, serotonin, prodromal

## Abstract

Apathy is a common and under-recognized disorder that often emerges in the prodromal phase of Parkinsonian diseases. The mechanism by which this occurs is not known, but recent evidence from patients with established Parkinson’s disease suggests that serotonergic dysfunction may play a role. The integrity of the raphe serotonergic system can be assessed alongside dopaminergic basal ganglia imaging using the radioligand ^123^I-ioflupane, which binds both serotonin and dopamine transporters. To investigate the relative roles of these neurotransmitters in prodromal parkinsonism, we imaged patients with idiopathic rapid eye movement sleep behaviour disorder, the majority of whom will develop a parkinsonian disorder in future. Forty-three patients underwent brain imaging with ^123^I-ioflupane single photon emission computed tomography and structural MRI. Apathy was quantified using the Lille Apathy Rating Scale. Other clinical parkinsonian features were assessed using standard measures. A negative correlation was observed between apathy severity and serotonergic ^123^I-ioflupane signal in the dorsal raphe nucleus (r = −0.55, *P* < 0.001). There was no significant correlation between apathy severity and basal ganglia dopaminergic signal, nor between dorsal raphe signal and other neuropsychiatric scores. This specific association between apathy and raphe ^123^I-ioflupane signal suggests that the serotonergic system might represent a target for the treatment of apathy.

## Teaser

Apathy is a common and debilitating condition that often emerges during prodromal Parkinson's disease. Using neuroimaging in patients with REM sleep behaviour disorder, Barber *et al*. show that apathy in this prodromal population is related to serotonin depletion in the dorsal raphe nucleus, suggesting a mechanism for its expression and a potential target for therapeutic intervention.

## Introduction

Apathy is a common and debilitating condition characterized by motivational deficits that impair emotional, social and behavioural function (Ang *et al.*, 2017). Despite mounting evidence that apathy is distinct from other neuropsychiatric disorders such as depression, little evidence exists to guide treatment approaches specific to apathy (Kirsch-Darrow *et al.*, 2011).

Apathy is a prominent feature of parkinsonian disorders, where it causes a significant burden on patients and caregivers alike (Muhammed and Husain, 2016). The role of dopaminergic projections in motivation and reward has led to much focus on the relationship between dopamine loss and apathy in Parkinson’s disease (Chong, 2018). Whilst it is clear that dopamine does indeed modulate apathy in Parkinson’s disease, its contribution to the underlying aetiology is less certain and may depend upon disease stage (MacDonald *et al.*, 2013). Recent evidence from functional neuroimaging suggests that depletion of serotonin may be an equally important feature in the development of apathy in early disease (Maillet *et al.*, 2016).

The serotonergic neurons of the dorsal raphe nucleus (DRN) project extensively to forebrain limbic regions implicated in the pathogenesis of apathy (Hornung, 2003). The characteristic caudal to rostral spread of brainstem pathology in Parkinson’s disease means that when the DRN is affected, this tends to occur before disease affects the nigro-striatal dopaminergic system (Braak *et al.*, 2003). It is conceivable, therefore, that pathological changes here may lead to neuropsychiatric impairment, with corresponding serotonergic imaging deficits, during the prodromal phase of Parkinson’s disease. Patients with idiopathic rapid eye movement sleep behaviour disorder (RBD) present an opportunity to study this period, since the majority of RBD patients will go on to develop a parkinsonian disorder (Schenck *et al.*, 2013). We recently described a high prevalence of apathy in RBD (Barber *et al.*, 2018), but the relationship to serotonergic depletion has never been studied.

The compound ^123^I-ioflupane is commonly used in single photon emission computed tomography (SPECT) imaging in the investigation of parkinsonian disorders (Brooks, 2016). Because it binds to both dopamine and serotonin presynaptic transporters, its imaging signal reflects the predominant transporter type in any given region (Scheffel *et al.*, 1997; de Win *et al.*, 2005). This property has made it possible to assess serotonergic signal in the raphe nuclei, where few dopamine transporters exist, alongside dopamine transporter binding in the basal ganglia (Qamhawi *et al.*, 2015). Here, we used this approach to investigate the neurochemical basis of apathy in RBD. We quantified apathy using the Lille Apathy Rating Scale (LARS), which provides a comprehensive assessment of apathy and can distinguish apathy from depression and cognitive impairment in patients with RBD (Sockeel *et al.*, 2006; Barber *et al.*, 2018). By examining both dopaminergic and serotonergic regions and exploring a range of neuropsychiatric features, we sought to describe the specific contribution of serotonergic loss to the development of apathy in this key prodromal population.

## Materials and methods

### Participants

The study was approved by the local ethical committee and written, informed consent was obtained from all participants.

Forty-three patients with idiopathic RBD volunteered for participation from an established cohort (Barber *et al.*, 2017). RBD was diagnosed by polysomnography according to International Classification of Sleep Disorders criteria (American Academy of Sleep Medicine, 2014). In all cases, RBD was idiopathic, not secondary to another neurological disorder or antidepressant medication. All patients were examined on the day of imaging to exclude the presence of clinical parkinsonism. In the eight participants taking antidepressant medication, this was withheld for at least 24 h prior to imaging.

### Clinical assessments

Apathy was assessed using the LARS, with a cut-off of >−22 defining apathetic patients in dichotomized analyses (Sockeel *et al.*, 2006). Depression was assessed using the Beck Depression Inventory II (BDI); anxiety using the Hospital Anxiety and Depression Scale (HADS); and cognition using the Montreal Cognitive Assessment (MoCA). Early motor impairment was quantified using part III of the Movement Disorders Society Unified Parkinson’s Disease Rating Scale (MDS-UPDRS); daytime sleepiness with the Epworth Sleepiness Scale; and olfaction using the Sniffin’ Sticks 16-item odour identification test. Orthostatic hypotension was measured as the difference between systolic blood pressure measured after lying flat for 3 min, and after standing for 2 min (see Barber *et al.*, 2017 for references to the above scales).

### SPECT/CT and MRI acquisition

See the online Supplementary material for details of image acquisition parameters.

### SPECT/CT analysis

^123^I-ioflupane SPECT scans were rigid-registered to each individual’s structural MRI using mutual information as similarity measure. Structural magnetic resonance images were transformed into 1 mm^3^ MNI152 space with FMRIB’s non-linear image registration tool (FNIRT) (Jenkinson *et al.*, 2012). Using the parameters determined from MRI, individual SPECT scans were then transformed into MNI152 space.

**Figure 1:**
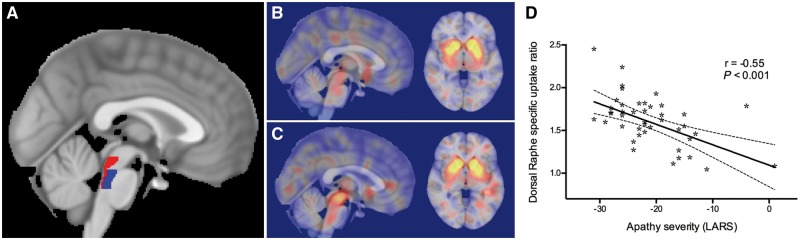
**Association between apathy severity and ^123^I-ioflupane SPECT/CT signal in the DRN.** (**A**) Standard space T_1_-weighted MRI template with regions of interest for the dorsal (red) and median (blue) raphe nuclei from the Harvard Ascending Arousal Network Atlas overlaid. (**B**–**C**) Illustrative example images from two patients with RBD, with SPECT/CT images registered to standard space and overlaid on the standard MRI template. Note the marked difference in signal within the brainstem between the two patients (sagittal images, *left*), despite similar signal in the basal ganglia (axial images, *right*). (**D**) A significant correlation is demonstrated between apathy severity, measured by the LARS, and ^123^I-ioflupane SPECT/CT signal in the DRN. Dashed lines indicate the 95% confidence interval of the best fit line.

All regions of interest were defined in MNI152 space (Supplementary Figs 1–7). Regions of interest for the dorsal raphe nucleus, median raphe nucleus and ventral tegmental area were obtained from the Harvard Ascending Arousal Network Atlas (Fig. 1) (Edlow *et al.*, 2012). Regions for the caudate, putamen and accumbens nuclei were derived from the Harvard-Oxford subcortical structural atlas. A substantia nigra region was drawn using an in-house template derived from neuromelanin-sensitive MRI. A background reference region in the superior lateral occipital cortex was defined using the Harvard-Oxford cortical structural atlas.

Normalized specific uptake ratios for each region of interest were calculated as the mean SPECT/CT value of voxels within that region divided by the mean SPECT/CT value of voxels within the reference region.

### Statistical analysis

Comparisons of variables between apathetic and non-apathetic patients were made using an independent samples *t*-test for continuous variables, and chi-square test for categorical variables. Pearson coefficients were used to correlate imaging and clinical variables. Linear regression was used to compare the relative effects of apathy, depression and cognitive impairment on dorsal raphe ^123^I-ioflupane signal. Statistical significance was considered as *P* < 0.05, with Bonferroni thresholds for multiple comparisons indicated where applicable. All analyses were carried out in SPSS version 24 (IBM).

### Data availability

Access to the data that support the findings of this study may be requested by application to the Oxford Parkinson’s Disease Centre Data Access Committee. Initial enquiries can be made to the corresponding author.

## Results

### Clinical characteristics

The mean age of patients was 65.1 years [standard deviation (SD) 7.60] and 42 patients were male. This high male to female ratio is representative of prevalent polysomnographically-diagnosed RBD (Barber *et al.*, 2017). The mean duration of RBD symptoms was 8.7 years (SD 7.34) and the mean time from polysomnographic diagnosis was 2.5 years (SD 2.27).

The mean LARS score was −21.0 (SD 6.69) and 18 patients (42%) scored in the apathetic range of the LARS. Since apathy itself might be a marker of emerging Parkinsonism, we compared a number of other clinical variables according to apathy status to investigate whether apathetic patients had more extensive signs of prodromal disease (Table 1). Postural hypotension was more severe in non-apathetic patients, but the significance of this did not reach the Bonferroni-adjusted threshold of *P* < 0.006. There was no difference in the degree of depression, anxiety, cognitive impairment, hyposmia, daytime sleepiness, or the rate of antidepressant use between apathetic and non-apathetic patients. These data suggest that apathetic patients on average were not at a more advanced prodromal stage than non-apathetic patients.

**Table 1:** Comparison of clinical variables between apathetic and non-apathetic RBD patients

Variable	Non-apathetic patients *n* = 25	Apathetic patients *n* = 18	*P*-value
**Clinical variables**			
Age, mean (SD), years	66.5 (6.92)	63.1 (8.18)	0.15
Male, *n* (%)	24 (96)	18 (100)	0.39
Number treated with antidepressants, (% of group)	3 (12)	5 (28)	0.19
Depression, mean (SD), BDI score	8.6 (7.15)	10.2 (10.57)	0.57
Cognition, mean (SD), MoCA score	26.0 (2.72)	24.9 (3.06)	0.26
Orthostatic systolic hypotension, mean (SD), mmHg	−10.6 (11.56)	−2.1 (15.06)	**0.04**
Olfaction, mean (SD), Sniffin Sticks score	7.0 (3.71)	7.7 (3.30)	0.50
Motor impairment, mean (SD), MDS-UPDRS III score	5.2 (4.08)	3.5 (2.57)	0.14
Anxiety, mean (SD), HADS score, anxiety component	5.76 (4.25)	5.2 (4.52)	0.67
Daytime sleepiness, mean (SD), ESS score	7.2 (4.77)	5.9 (4.61)	0.41
**^123^I-ioflupane SPECT/CT signal by region of interest**			
Region of interest specific uptake ratio			
DRN, mean (SD)	1.70 (0.259)	1.45 (0.277)	**0.005**
Median Raphe nucleus, mean (SD)	1.79 (0.344)	1.71 (0.293)	0.42
Ventral tegmental area, mean (SD)	2.35 (0.536)	2.28 (0.581)	0.67
Right accumbens nucleus, mean (SD)	3.74 (0.393)	3.94 (0.792)	0.34
Left accumbens nucleus, mean (SD)	4.26 (0.467)	4.40 (0.676)	0.45
Right caudate nucleus, mean (SD)	2.84 (0.383)	2.89 (0.417)	0.70
Left caudate nucleus, mean (SD)	3.09 (0.377)	3.07 (0.389)	0.90
Right putamen, mean (SD)	4.21 (0.381)	4.18 (0.686)	0.84
Left putamen, mean (SD)	4.10 (0.462)	4.31 (0.693)	0.24
Right substantia nigra, mean (SD)	2.29 (0.394)	2.12 (0.545)	0.24
Left substantia nigra, mean (SD)	2.11 (0.353)	2.11 (0.539)	1.0

BDI = Beck Depression Inventory II; ESS = Epworth Sleepiness Scale; HADS = Hospital Anxiety and Depression Scale; MoCA = Montreal Cognitive Assessment.

### Imaging data

Apathetic patients had significantly lower ^123^I-ioflupane signal in the DRN than non-apathetic patients (mean 1.45 versus 1.70, *P* = 0.005), whereas the ^123^I-ioflupane signal did not differ in any other region of interest (Table 1). Across all patients there was a significant negative correlation between apathy severity and ^123^I-ioflupane signal in the DRN (r = −0.55, *P* < 0.001), indicating that greater apathy was associated with reduced serotonin signal (Fig. 1D). To exclude a possible confounding effect of antidepressant medication, we conducted a sensitivity analysis excluding the eight participants treated with antidepressant medication. The same association between apathy and DRN signal was observed (r = −0.51, *P* = 0.002, Supplementary Fig. 8).

To ensure the association was not merely an artefact of signal spillover from neighbouring regions, we performed a multiple regression analysis including ^123^I-ioflupane signal from the nearby midbrain peak as a covariate. This strengthened further the association between apathy and DRN signal (Supplementary material).

To investigate whether the association between apathy and DRN signal was explained by a general reduction in brain monoamine transporters in apathetic patients, we looked at the relationship between apathy and ^123^I-ioflupane signal in the median raphe nucleus and a number of dopaminergic regions (Table 2). LARS scores did not correlate significantly with ^123^I-ioflupane signal in any of these regions. This specific association between apathy and signal in the DRN suggests that apathy is not simply a marker of generalized prodromal neurodegeneration.

**Table 2:** Correlation between apathy severity and ^123^I-ioflupane SPECT/CT signal in regions of interest

Region of interest specific uptake ratio	Pearson correlation coefficient with apathy severity (LARS) across all patients (*n* = 43)	*P*-value
Dorsal raphe nucleus	−0.55	**<0.001**
Median raphe nucleus	−0.25	0.11
Ventral tegmental area	−0.02	0.91
Right accumbens nucleus	0.18	0.28
Left accumbens nucleus	0.16	0.30
Right caudate nucleus	0.12	0.46
Left caudate nucleus	0.17	0.27
Right putamen	0.07	0.66
Left putamen	0.17	0.27
Right substantia nigra	−0.02	0.88
Left substantia nigra	−0.08	0.62

We next assessed the degree to which the relationship between DRN serotonin signal and apathy was dissociable from other parkinsonian symptoms. Of particular interest were comparisons with depression and cognitive impairment, respectively, as these syndromes exhibit considerable phenotypic overlap with apathy. Table 3 shows correlations between signal in the DRN and secondary clinical measures of interest. None of these correlations reached significance (Bonferroni-adjusted significance threshold, *P* < 0.006). Signal in the dorsal raphe did not correlate with age (r = 0.02, *P* = 0.92).

**Table 3:** Correlation between dorsal raphe SPECT/CT signal and clinical variables

Variable	Correlation with DRN across all patients (*n* = 43)	*P*-value
Apathy (LARS score)	−0.55	**<0.001**
Depression (BDI score)	−0.25	0.10
Anxiety (HADS score)	−0.07	0.66
Cognition (MoCA score)	0.25	0.11
Motor impairment (MDS-UPDRS III score)	0.19	0.22
Daytime sleepiness (ESS score)	−0.10	0.51
Orthostatic systolic BP drop	−0.27	0.08
Olfaction (Sniffin Sticks score)	−0.08	0.65
Age	0.02	0.92

BDI = Beck Depression Inventory II; BP = blood pressure; ESS = Epworth Sleepiness Scale; HADS = Hospital Anxiety and Depression Scale; MoCA = Montreal Cognitive Assessment.

To further dissociate the closely related neuropsychiatric features of apathy, depression and cognitive impairment, we used multiple linear regression, with dorsal raphe ^123^I-ioflupane signal as the dependent variable and LARS, BDI and MOCA scores as predictors. Only LARS scores had a significant effect on dorsal raphe signal (beta = −0.50, *P* = 0.001), whereas BDI scores (beta = −0.17, *P* = 0.20) and MOCA scores (beta = −0.09, *P* = 0.51) did not. Thus apathy, but not depression or cognitive function, appeared to be significantly and specifically associated with ^123^I-ioflupane signal in the DRN.

## Discussion

In this study, we demonstrate for the first time a relationship between serotonin loss in the DRN and clinical apathy in patients with RBD, a key population representing the prodromal phase of Parkinson’s disease and related alpha-synucleinopathies. Serotonergic dysfunction is associated with apathy early in Parkinson’s disease (Maillet *et al.*, 2016); our data extend this important finding by revealing that this association emerges prior to the onset of clinical parkinsonism. Importantly, neither depression, anxiety, nor cognition correlated with the DRN serotonin signal, further emphasizing apathy as a distinct neuropsychiatric entity.

A key dissociation was also seen between DRN serotonergic signal and dopaminergic binding in the basal ganglia. The lack of correlation between dopaminergic signal and apathy not only implies that serotonergic degeneration might be a key factor in the pathogenesis of apathy, but also suggests that the association is not explained simply by more advanced prodromal neurodegeneration in apathetic patients. This assertion is further supported by the finding that apathetic patients did not have more severe clinical markers of evolving parkinsonism than non-apathetic patients.

While patients with idiopathic RBD can offer an unprecedented insight into the early stages of parkinsonian disorders, they are a heterogeneous population. Around two-thirds are likely to develop Parkinson’s disease or dementia with Lewy bodies, while a smaller number will convert to multiple system atrophy (Schenck *et al.*, 2013). In some cases, however, this may occur more than a decade from RBD onset, and a minority of individuals may never develop a neurodegenerative disorder (Iranzo *et al.*, 2017). The fact that an association between DRN serotonergic signal and apathy was observed despite this heterogeneity suggests that the role of serotonin in apathy might be a particular feature of patients with RBD. One possible explanation for this is the close anatomical proximity of the raphe nuclei to the locus coeruleus/subcoeruleus complex, an important player in the regulation of REM sleep. The arrival of ascending neurodegeneration in tandem in these neighbouring structures during prodromal disease would be consistent with Braak’s hypothesis of Parkinson’s pathology (Braak *et al.*, 2003), and it may be that the extent to which this occurs underlies the correlation between raphe signal and apathy observed in RBD.

Our findings imply that this serotonin-dependent apathy is not a harbinger of emerging parkinsonism, but rather an example of the way in which the differential involvement of neurotransmitter systems among patients, due to subtle variations in the anatomical pattern of disease, might explain some of the phenotypic variation observed in synucleinopathies. In patients with established Parkinson’s disease, for example, it is known that motor signs relate to nigro-striatal dopamine loss (Benamer *et al.*, 2000), whilst noradrenergic pathways are implicated in the development of postural hypotension (Sommerauer *et al.*, 2018). Our data suggest that patients with a greater burden of disease in serotonergic pathways may represent a subtype prone to developing apathy.

Although no other studies have examined the relationship between serotonergic signalling and apathy in RBD, the possibility that disruption in these pathways affects a subtype of apathetic patients rather than being a general marker of neurodegeneration is supported by a recent study of patients with early Parkinson’s disease, which used PET with multiple tracers to further dissociate the roles of serotonin and dopamine (Maillet *et al.*, 2016). No differences were observed in serotonin signal between non-apathetic patients with Parkinson’s disease and control subjects, despite significantly reduced dopaminergic uptake in the Parkinson’s disease group. Within Parkinson’s disease patients, however, reduced serotonin signal was associated with the presence of apathy.

Apathy may not be the only consequence of raphe serotonergic degeneration. Using similar methodology to ours in a large Parkinson’s disease cohort, Qamhawi *et al.* (2015) also demonstrated that serotonin depletion affected a subgroup of patients and was dissociable from dopaminergic degeneration. Apathy was not assessed, but an association was found between raphe signal and tremor severity. Given that the whole raphe complex was included in their region of interest, this result may reflect the role of other subpopulations of serotonergic neurons, including more caudal projections to the cerebellum and deep brain nuclei implicated in the pathogenesis of tremor.

It is unlikely that serotonin signalling alone underlies the complex clinical syndrome of apathy in parkinsonian disorders. Exogenous dopamine (or dopamine receptor agonists) can not only improve the symptoms of apathy, but also modulate objective measures of reward sensitivity, a key component of motivation for action (Thobois *et al.*, 2013; Muhammed *et al.*, 2016). It is possible that the differential contributions of serotonin and dopamine loss to the expression of apathy in Parkinson’s disease may vary according to disease stage, with mesolimbic dopaminergic pathways affected later than the raphe serotonergic system. This might have important implications for the treatment of apathy in patients with prodromal and early Parkinson’s disease, where serotonergic agents could conceivably have greater efficacy. Interventional studies testing such hypotheses must be a near-term priority since little evidence exists to guide the specific treatment of apathy.

Our study has a number of notable strengths. Investigating patients with polysomnographically-proven RBD provides unparalleled enrichment for cases of prodromal parkinsonism. At this early stage, when neurodegeneration is largely confined to the brainstem, the confounding effects of more widespread degeneration involving other neurotransmitter systems is limited compared to established Parkinson’s disease patients.

The CT-based attenuation correction that we used for SPECT/CT processing greatly enhanced the precision with which we could analyse brainstem structures. The accuracy of our brainstem nuclei measurements was further enhanced by the use of histologically derived regions of interest, giving us the best possible representations of their true anatomy.

These methodological strengths help to address one of the main limitations of our study: that the nuclei studied are small and therefore close to the resolution of our SPECT imaging. A second limitation is the use of a relatively non-specific SPECT ligand, meaning that our assessment of the serotonergic system is limited to regions that do not contain significant numbers of dopamine transporters. Although the lack of a separate control group could be considered a limitation, the primary aim of our study was to explore the neural basis for the phenotypic heterogeneity that occurs amongst RBD patients, rather than to show how they differ from control subjects. It is interesting to note, however, that control subjects rarely exhibit apathy (Barber *et al.*, 2018), despite having similar levels of raphe serotonin transporters to patients with Parkinson’s disease (Qamhawi *et al.*, 2015). One possible explanation for this apparent discrepancy would be that individuals may have different levels of intrinsic reserve in the raphe serotonergic system, which might lead to different levels of clinical apathy only when the system is exposed to a neurodegenerative insult.

In summary, we have shown that apathy is associated with serotonergic depletion in the dorsal raphe nucleus in patients with idiopathic RBD, suggesting that early degeneration in this area might underlie the development of apathy during the prodromal phase of Parkinson’s disease. The specific role of serotonin is evidenced by the fact that this relationship is dissociable from dopaminergic degeneration and other neuropsychiatric features, and should prompt investigation of serotonergic agents in the treatment of apathy, even in the absence of depression.

## Supplementary Material

Supplementary Materials

## Abbreviations

DRN: dorsal raphe nucleus
LARS: Lille Apathy Rating Scale
RBD: rapid eye movement sleep behaviour disorder
SPECT/CT: single photon emission computed tomography with CT attenuation correction
