# Supplementary material for: Apathy in rapid eye movement sleep behaviour disorder is associated with serotonin depletion in the dorsal raphe nucleus
Source: Brain. 2018 Sep 12;141(10):2848–54. doi: 10.1093/brain/awy240 (PMC6158712; doi:10.1093/brain/awy240)
Supplement: Supplementary Materials [file awy240_supplementary_materials.pdf]

Supplementary Materials

SPECT/CT acquisition protocol

Subjects were injected with 185 MBq +/-10% of ^{123}I-ioflupane (provided as DaTscan™ injection, GE Healthcare). Potassium iodide 120mg was administered one hour prior to, and 24 hours after, injection of ^{123}I-ioflupane to block thyroid uptake. SPECT/CT images were acquired three hours post injection on a Discovery 670 gamma camera (GE Healthcare, Haifa).

SPECT parameters: 120 projections, 30 seconds per projection, 128 x 128 matrix. CT parameters: 16 slice, helical acquisition, 120 KV, 40 mA, noise index 30. The SPECT/CT data was reconstructed using HERMES Hybrid Recon (HERMES Medical Solutions, AB Stockholm) OSEM, 15 iterations, 4 subsets with attenuation correction from CT, collimator resolution recovery, and Monte Carlo scatter correction. The isotropic voxel size of reconstructed images was 2.21 mm^3.

MRI acquisition protocol

Structural MRI was acquired to facilitate registration of SPECT/CT images to standard space. MRI scanning was performed as follows using a 3T Siemens Trio (Erlangen, Germany): 12-channel receive-only head coil, MPRAGE, TE/TR/TI=4.7ms/2040ms/900ms; 192 axial slices; isotropic voxel size 1mm^3; 6 minutes.

Supplementary figures demonstrating regions of interest used in SPECT/CT analysis

Figure S1. Superior lateral occipital cortex (Harvard-Oxford Cortical Structural Atlas)

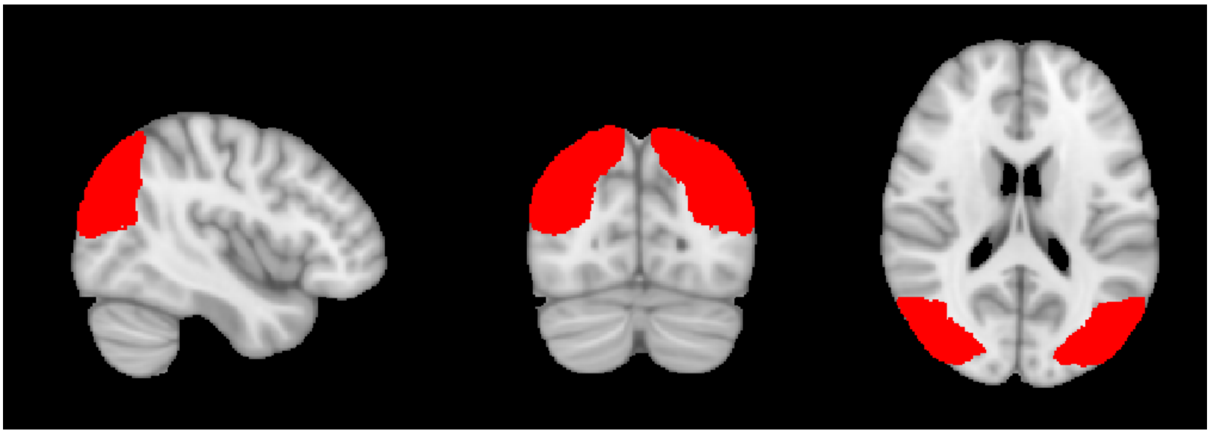

Image MNI co-ordinates: 43, -76, 18

The above region was used as the background reference region in order to calculate specific uptake ratios for the regions of interest below. We used this region because it is low in monoamine transporters, distant from any signal from the regions of interest studied, and had full MRI and SPECT coverage in all of our participants.

Figure S2. Substantia nigra (in-house template). Right = red; Left = blue

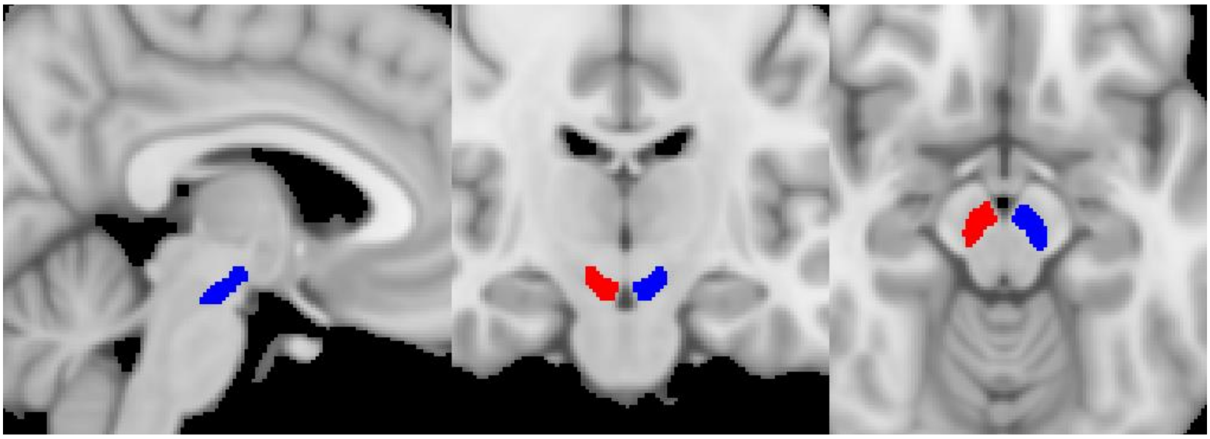

Image MNI co-ordinates: -4, -18, -14

Figure S3. Putamen (Harvard-Oxford Subcortical Structural Atlas). Right = red; Left = blue

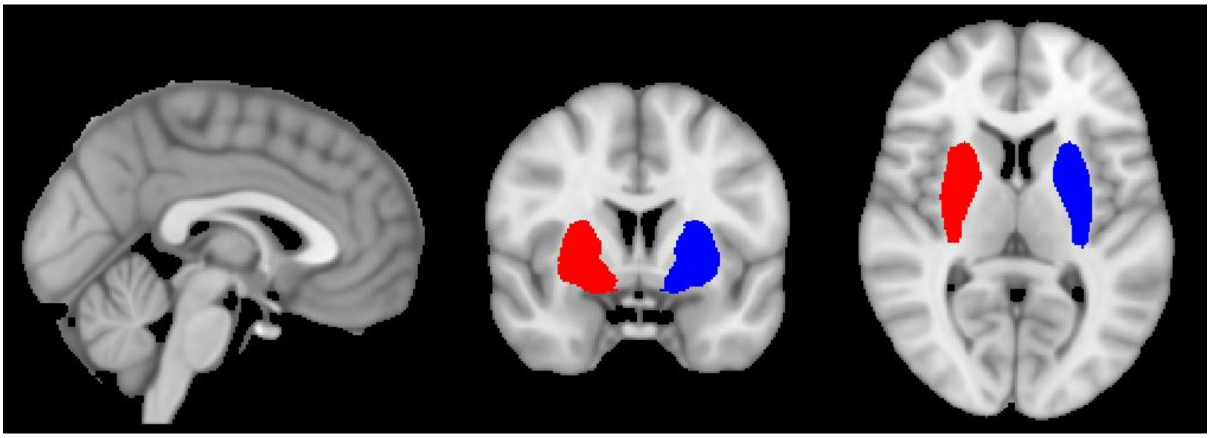

Image MNI co-ordinates: 0, 3, 7

Figure S4. Caudate nuclei (Harvard-Oxford Subcortical Structural Atlas). Right = red; Left = blue

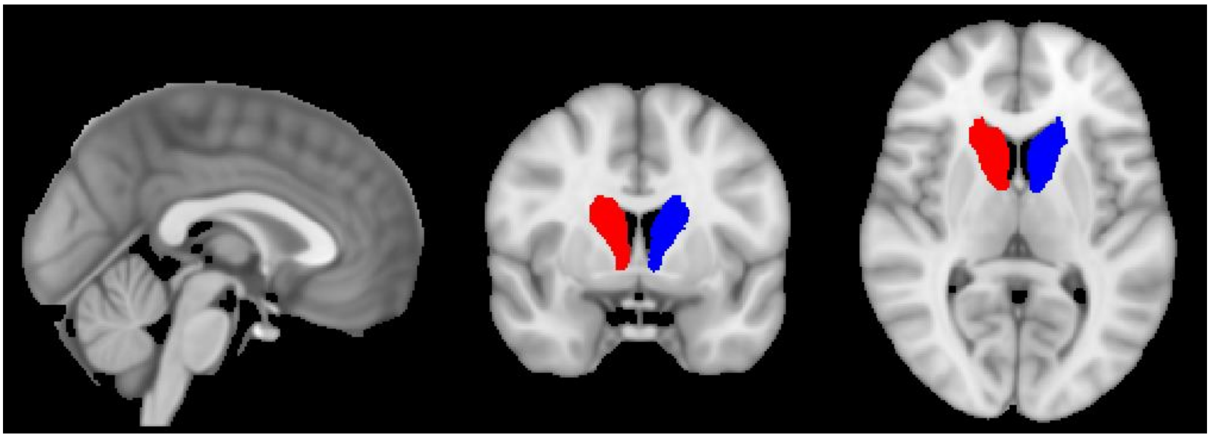

Image MNI co-ordinates: 0, 3, 7,

Figure S5. Accumbens nuclei (Harvard-Oxford Subcortical Structural Atlas). Right = red; Left = blue

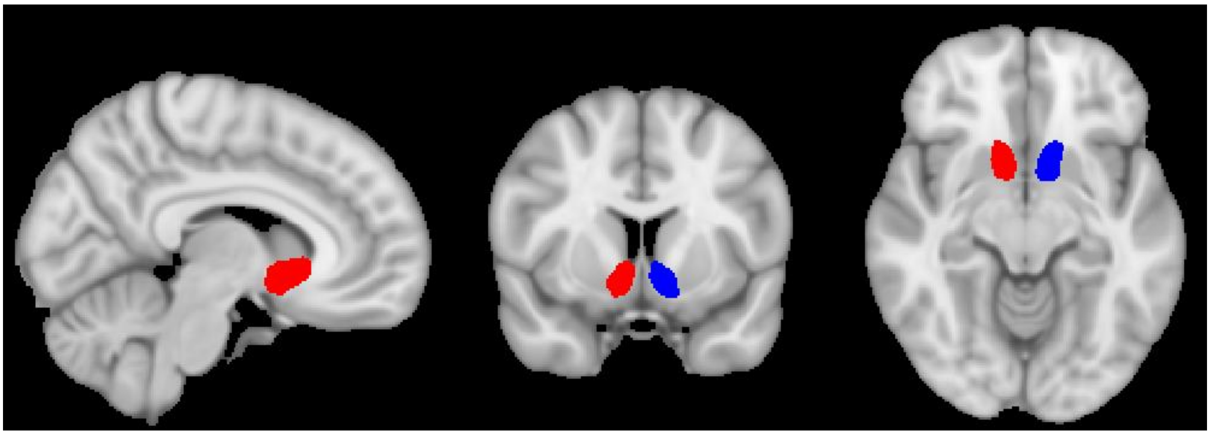

Image MNI co-ordinates: 8, 8, -10

Figure S6. Ventral tegmental area (Harvard Ascending Arousal Network Atlas)

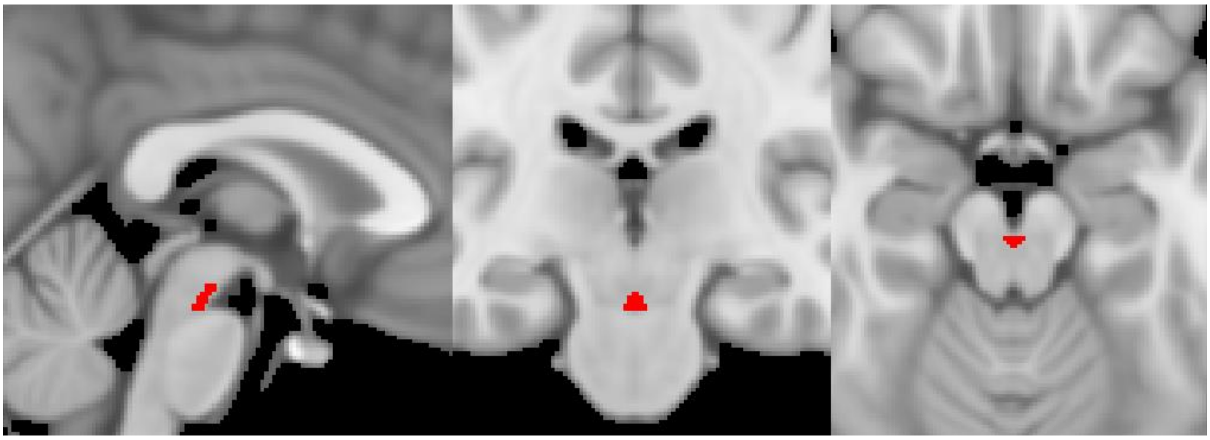

Image MNI co-ordinates: 0, -24, -18

Figure S7. Raphe nuclei (Harvard Ascending Arousal Network Atlas). Red = dorsal raphe nucleus, blue = median raphe nucleus

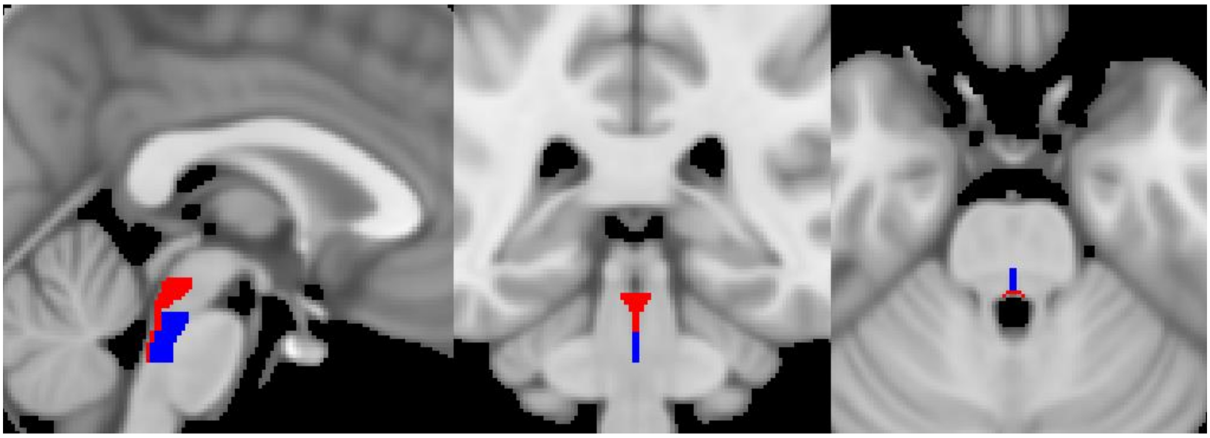

Image MNI co-ordinates: 0, -35, -28

<u>Figure S8.</u>

Sensitivity analysis showing the correlation between apathy severity and 123I-ioflupane SPECT signal in the dorsal raphe nucleus excluding the 8 participants taking antidepressant medication.

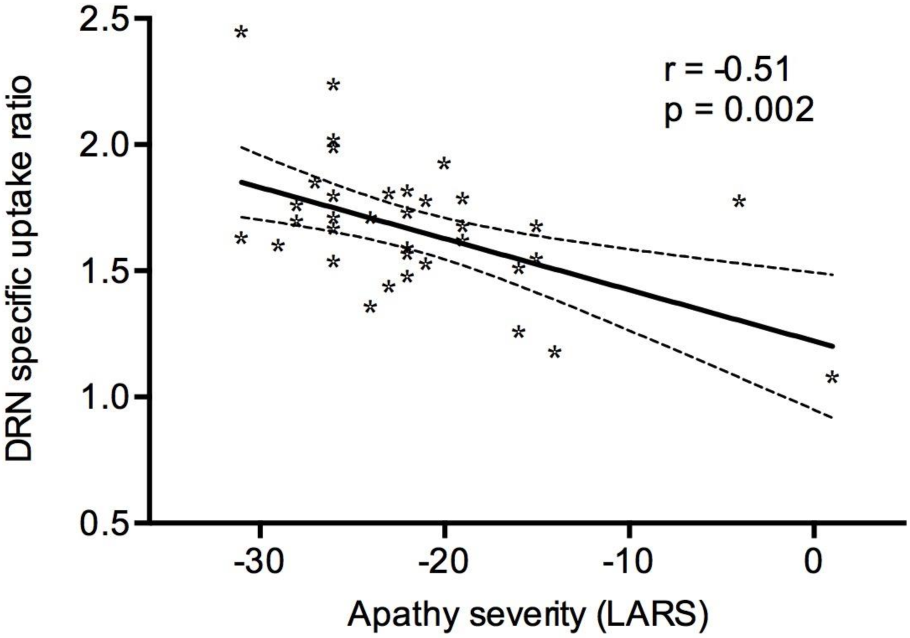

Adjusting for signal spillover from surrounding regions

Figure S9 below shows a midline sagittal image of the group average ioflupane signal, overlaid on a standard space structural MRI image. It can be seen that the area of peak signal in the brainstem is situated largely in the midbrain, anterior and rostral to the DRN region illustrated above (figure S7). In order to ensure that the signal extracted from our DRN was not influenced by spillover from this nearby peak, we calculated the mean signal from this region, shown in figure S10. We then undertook a multiple regression analysis with apathy severity as the dependent variable and signal from this region as a covariate, along with the DRN region of interest.

Figure S9: midline sagittal view of the average ioflupane signal across all participants

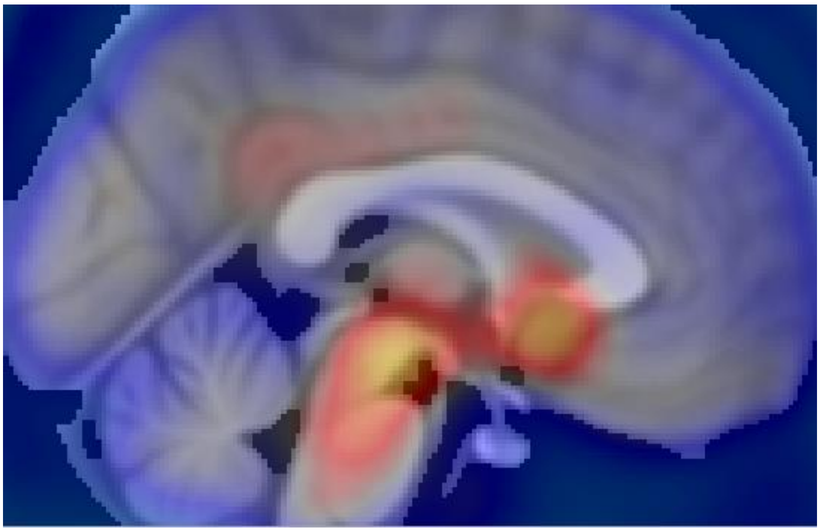

Figure S10: region of interest representing the peak voxels from the area of high signal in the midbrain is shown in green.

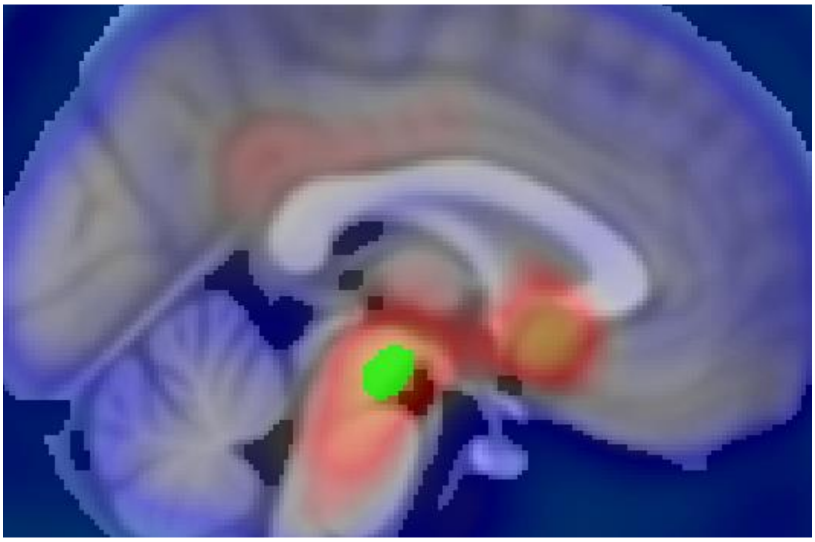

There was no direct correlation between the midbrain peak region and apathy severity (r = -0.02, p = 0.88).

In the multiple regression analysis including the DRN and midbrain peak ROIs as covariates, the association between lower DRN signal and increased apathy severity was stronger (beta = -0.70, p<0.001) than seen with the simple correlation analysis. This is because the effect of the midbrain peak ROI in the regression analysis was in the opposite direction, with *increased* signal in this region predicting greater apathy severity (beta = 0.32, p = 0.03).

Given the univariate result and biological considerations, it is unlikely that preserved dopaminergic transmission leads to more severe apathy. The reason for this apparent discrepancy can be deduced from the regression equation:

$$LARS_{est} = -4.399 - 16.217 \times DRN + 3.873 \times midbrain\ peak$$

The net result is that spillover of signal from the midbrain to the DRN, albeit numerically small, leads to underestimation of the raphe serotonergic deficit, and the model predictor is adjusted accordingly. Thus, we can conclude that the association seen between DRN signal and apathy severity is indeed not caused by spill-over from the adjacent high signal region in the midbrain.
